# Visceral Leishmaniasis in a 25-Year-Old Female Kidney Transplant Recipient from a Non-Endemic Region: A Case Report from Romania

**DOI:** 10.3390/microorganisms13020403

**Published:** 2025-02-12

**Authors:** Alin Gabriel Mihu, Mariana Patiu, Delia Monica Dima, Daniela Adriana Oatis, Cristina Mihaela Cismaru, Rodica Lighezan, Tudor Rares Olariu

**Affiliations:** 1 Center for Diagnosis and Study of Parasitic Diseases, Department of Infectious Disease, Victor Babes University of Medicine and Pharmacy, 300041 Timisoara, Romania; alin.mihu@umft.ro (A.G.M.); rolariu@umft.ro (T.R.O.); 2 Department of Biology and Life Sciences, Vasile Goldis Western University, 310300 Arad, Romania; 3 Department of Hematology, Ion Chiricuta Oncology Institute, 400015 Cluj-Napoca, Romania; mpatiu@yahoo.com (M.P.); delia.dima@iocn.ro (D.M.D.); 4 Department of Medicine, Vasile Goldis Western University, 310300 Arad, Romania; 5 Clinical Hospital of Infectious Diseases of Cluj-Napoca, 400348 Cluj-Napoca, Romania; cristina.cismaru@umfcluj.ro; 6 Department of Infectious Diseases, Iuliu Hatieganu University of Medicine and Pharmacy, 400348 Cluj-Napoca, Romania; 7 Discipline of Parasitology, Department of Infectious Disease, Victor Babes University of Medicine and Pharmacy, 300041 Timisoara, Romania; 8 Regional Blood Transfusion Center, 300737 Timisoara, Romania; 9 Clinical Laboratory, Municipal Clinical Emergency Teaching Hospital, 300254 Timisoara, Romania; 10 Patogen Preventia, 300124 Timisoara, Romania

**Keywords:** leishmaniasis, kidney transplant, Romania

## Abstract

Visceral leishmaniasis is a rare parasitic infection in non-endemic regions such as Romania. We report the case of a 25-year-old female kidney transplant recipient from Cluj County, Romania, who developed persistent bicytopenia with anemia and thrombocytopenia. Despite no history of travel outside Cluj County and being the only organ recipient from the same donor to experience signs and symptoms, she was diagnosed with visceral leishmaniasis. The second bone marrow aspirate performed revealed *Leishmania* amastigotes. She was quickly sent to Victor Babes Infectious Disease Hospital in Bucharest for additional tests and treatment. The kidney function of the patient was maintained. This case highlights the importance of considering leishmaniasis in immunosuppressed patients presenting with unexplained cytopenia, even in non-endemic regions. This is the first documented case of visceral leishmaniasis in a kidney transplant recipient in Romania. The present report could serve as a foundation for future educational programs targeted toward informing both healthcare providers and patients about the risks, diagnosis, and management of leishmaniasis in immunosuppressed individuals in non-endemic regions.

## 1. Introduction

*Leishmania* is an obligate intracellular protozoan parasite from the *Trypanosomatidae* family, transmitted by phlebotomine sand flies. This parasite causes cutaneous, mucocutaneous, and visceral leishmaniasis (VL), affecting populations in both the old and new world. The disease is characterized as complex, with multiple subspecies leading to diverse clinical presentations, often complicating the diagnosis process and the management of clinicians [1,2].

Leishmaniasis is a neglected tropical and subtropical disease that disproportionately affects the world’s poorest populations in over 90 countries across Asia, Africa, the Middle East, and Central and South America. Although likely underreported, it is estimated that between 700,000 and 1.2 million cases of cutaneous leishmaniasis occur annually, with about 95% of these cases found in the Americas, the Mediterranean basin, the Middle East, and Central Asia. In contrast, the incidence of VL has significantly declined from earlier estimates of 400,000 cases per year to fewer than 100,000. Over 95% of VL cases reported to the World Health Organization [3] come from countries such as Brazil, China, Ethiopia, India, Kenya, Nepal, Somalia, and Sudan. Risk factors for leishmaniasis include poverty, population displacement, malnutrition, poor living conditions, and immunosuppression [4,5,6,7].

VL, also referred to as kala-azar in certain regions, is the most severe and life-threatening form of leishmaniasis. It is primarily caused by *Leishmania donovani* in East Africa and India and *Leishmania infantum* in the Mediterranean, the Middle East, and the Americas. In some cases, other species more commonly linked to cutaneous leishmaniasis can cause VL, particularly in people with weakened immune systems [8,9].

Leishmaniasis is commonly transmitted to humans through the bite of a blood-feeding phlebotomine sandfly [10]. Alternative infection routes for leishmaniosis were reported particularly in regions where cases were diagnosed despite the absence of confirmed vectors or any described *Phlebotominae* species. Proven non-vectorial transmission routes in humans include organ transplantation, transplacental or congenital transmission, blood transfusion, and the sharing of needles among drug users [11,12,13].

Symptoms develop gradually in about 5% of infected individuals, often months or even years after the initial infection. Without treatment, VL can progress to complications such as bone marrow failure, extreme wasting, and severe bleeding [14]. Immunosuppressed individuals, including those with HIV or on immunosuppressive therapies, face an even higher risk of death [15]. The disease typically presents with a combination of prolonged fever, weight loss, enlarged liver and spleen, pancytopenia, and hypergammaglobulinemia [16].

Although Romania is traditionally considered nonendemic for leishmaniasis, recent cases highlight its emerging presence [17], particularly considering climate change and increased international travel. Cluj County, situated at the crossroads of historical and environmental factors, offers a unique perspective on the spread of this vector-borne disease.

This case report aimed to shed light on diagnostic challenges, clinical presentation, and treatment outcomes, contributing to the growing body of knowledge on leishmaniasis in non-endemic regions. It also emphasizes the importance of awareness among healthcare providers, advocating for improved diagnostic tools and surveillance systems, especially in patients with febrile pancytopenia, in order to prevent underdiagnosis and mismanagement of the disease. Ultimately, this report could serve as a valuable reference for both local and global efforts to understand and combat leishmaniasis.

## 2. Case Presentation

**Figure 1:**
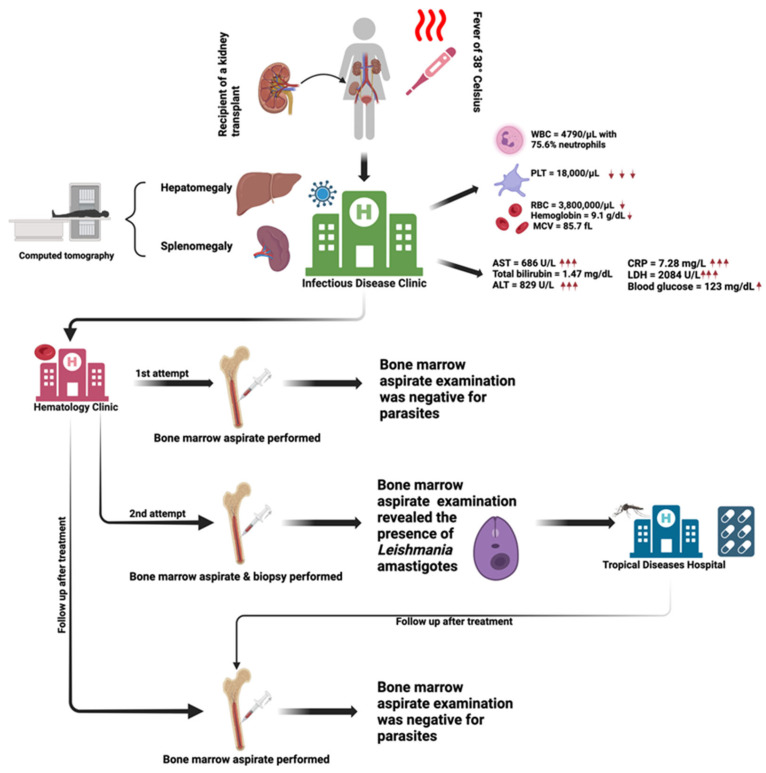
Schematic representation of the diagnostic pathway for a 25-year-old female renal transplant recipient with bicytopenia and febrile syndrome who presented to the Infectious Disease Clinic in Cluj-Napoca, Romania. The patient was transferred to the Hematology Clinic in Cluj-Napoca for bone marrow aspirates on two separate occasions, where a diagnosis of visceral leishmaniasis was established from the second aspirate, referred to the Tropical Diseases Hospital in Bucharest, Romania, for confirmation and treatment, and later returned to the Hematology Clinic in Cluj-Napoca for follow-up care.

A 25-year-old female patient who had received a kidney transplant from a brain-dead donor (following a traffic accident) presented to the Infectious Disease Clinic in Cluj-Napoca, Central Romania, for evaluation and treatment of bicytopenia and a persistent febrile syndrome (38 °C) accompanied by hepatosplenomegaly. She was also the only organ recipient from the donor who presented bicytopenia accompanied by signs and symptoms (Figure 1).

From the patient’s medical history, it is known that she was diagnosed with stage 5 chronic kidney disease (CKD), according to Kidney Disease Improving Global Outcomes (KDIGO), requiring dialysis. This condition developed one year prior to her presentation due to IgA nephropathy (confirmed by renal biopsy). She underwent dialysis for four months before receiving a kidney transplant and has since been on immunosuppressive therapy with Tacrolimus, Prednisone, and Mycophenolate Mofetil, which was suppressed in May 2016 due to cytopenia. The kidney transplant was performed nine months before her admission in October 2015.

The current illness was noticed by clinicians one month before hospitalization, during routine post-transplant investigations, when the patient was found to have bicytopenia (mild anemia with thrombocytopenia) accompanied by an elevated C-reactive protein (CRP).

A detailed medical history revealed no travel outside Romania or to the South-Eastern part of the country. The patient underwent clinical and laboratory investigations, which ruled out Epstein-Barr virus and Cytomegalovirus infection, with negative blood cultures. Tacrolimus was adjusted according to renal function by the nephrologist.

A hematology consultation was performed, along with a first bone marrow aspirate from the sternum. The slides obtained were stained with May-Grünwald Giemsa [18,19]. Microscopic examination of the bone marrow smears revealed the presence of myeloid cell lines without abnormalities. The cytopenia was considered to have a peripheral cause, and corticosteroid therapy was initiated (Medrol 48 mg). Despite this treatment, the patient’s condition worsened, and two weeks later, she continued to present recurrent fever episodes, prompting readmission to the Infectious Diseases Clinic. A thoraco-abdomino-pelvic CT scan revealed hepatosplenomegaly, a small pelvic fluid collection, and a right-sided pleural effusion. Treatment was initiated with antibiotics (imipenem/cilastatin), antivirals (ganciclovir), antifungals (voriconazole), granulocyte growth factor, and symptomatic medications. During her hospitalization, she received transfusions of 2 units of platelet concentrate and 2 units of fresh frozen plasma, with patient monitoring following the department’s protocol; no adverse effects were observed.

Following detailed investigations, the patient was transferred to the Hematology department with a suspected diagnosis of hemophagocytic syndrome. Upon admission, she presented with a compromised general condition, inspiratory dyspnea, oxygen saturation of 93% on room air, significant asthenia, and fatigue. Clinical examination revealed a conscious and cooperative patient with a fever of 38 °C, pallor of the skin and mucous membranes, no palpable superficial lymphadenopathy, a mature arterio-venous fistula in the left arm, a supple abdomen with normal respiratory movement, and splenomegaly extending 6–7 cm below the costal margin. Breath sounds were diminished in the lower half of the right hemithorax.

Laboratory tests revealed moderate normochromic, normocytic anemia, severe thrombocytopenia, significant hepatocellular injury (ALT > AST), mildly elevated total bilirubin, increased lactate dehydrogenase (LDH), elevated glucose, slightly raised alkaline phosphatase, hypocalcemia, and hypomagnesemia (Table 1 Initial Presentation).

Pharmacological treatment continued with antibiotics, antivirals, and antifungals corticosteroids (dexamethasone 12 mg), a gastric protector (proton pump inhibitor), loop diuretics, hepatoprotective agents, and symptomatic medications.

**Figure 2:**
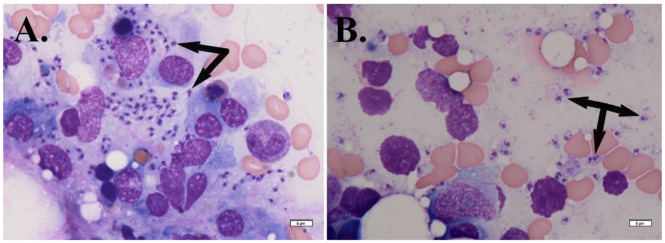
May-Grünwald Giemsa-stained bone marrow aspirate showing numerous *Leishmania* amastigotes (black arrows) both within macrophages (**A**) and freely dispersed outside the cells (**B**) (×400).

A secondary bone marrow aspirates (along with a trephine biopsy) was performed, and the smears presented with unspread and fatty tissue along with highly cellular bone marrow. The granulocytic series comprised roughly 70% of the nucleated cells, with hypergranularity and a left shift. The erythroid series was normoblastic, containing erythroblasts at all stages of maturation, and the megakaryocytic series showed no abnormalities. Amastigotes were identified both individually and in small clusters (Figure 2A,B), likely originating from ruptured macrophages (Figure 2A).

**Figure 3:**
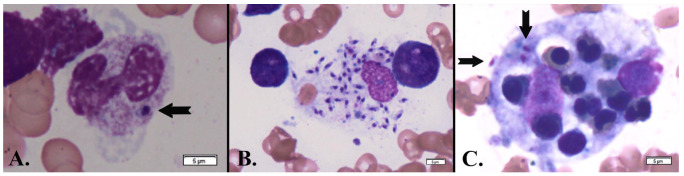
May-Grünwald Giemsa-stained bone marrow aspirate showing an unsegmented (band) neutrophil (**A**) that phagocytized a *Leishmania* amastigote (black arrows), numerous amastigotes within a reticular macrophage (**B**), and three amastigotes inside a macrophage undergoing hemophagocytosis of several normochromic erythroblasts (**C**) (×1000).

*Leishmania* amastigotes were also rarely noticed in band neutrophils (Figure 3A) but found frequently in reticular macrophages (Figure 3B). Significant hemophagocytosis by macrophages of erythroblasts was observed (Figure 3C). These findings, in combination with the patient’s clinical background (immunosuppression for kidney transplantation, bicytopenia, splenomegaly, and prolonged fever), strongly pointed to visceral leishmaniasis. Given the urgency, the patient was transferred under medical supervision to “Victor Babeș” Infectious and Tropical Diseases Clinical Hospital, Bucharest, for supplementary tests and appropriate treatment.

At the “Victor Babeș” Hospital, the diagnosis of visceral leishmaniasis was confirmed with polymerase chain reaction (PCR) [20], despite the absence of an epidemiological link, as the patient had no history of travel outside Romania or to the southeastern part of the country. Under specialized treatment, the patient showed favorable progress and was discharged with recommendations for weekly follow-ups at specialized outpatient clinics in Cluj County. She continued to be monitored by the Infectious Diseases, Hematology, and Nephrology services in Cluj-Napoca.

Two months after the confirmation of visceral leishmaniasis, the patient presented for a follow-up at the Hematology department. At the time of the consultation, her general condition had improved compared to previous evaluations. She was conscious, cooperative, and afebrile. Clinical examination revealed a Cushingoid facies and abdominal palpation detected a spleen palpable 3 cm below the costal margin. Laboratory tests (Table 1 Follow-up presentation), an abdominal ultrasound, and a repeat bone marrow aspirate were performed. The abdominal ultrasound showed mild splenomegaly, with the spleen measuring 14 cm in length and 7.9 cm in width.

The follow-up bone marrow aspirate examination revealed richly cellular smears consisting of tissue and bone marrow blood. All myeloid series were present: the erythroid series was normocytic with elements at all stages of maturation; the granulocytic series was well-represented and maturing; and the megakaryocytic series was observed in tissue areas. No *Leishmania* amastigotes or hemophagocytosis was noted. The patient was discharged with recommendations for weekly monitoring of her complete blood count. The kidney function was successfully preserved.

## 3. Discussion

Bicytopenia, characterized by the reduction of any two major blood cell lineages (erythrocytes, leukocytes, or platelets), is an important hematological indicator with diverse etiologies. Bicytopenia can be caused by a broad spectrum of causes, ranging from transient marrow suppression due to viral infections to infiltrative malignancies [21,22]. Nutritional deficiencies, such as vitamin B12 and folate deficiency, have also been extensively documented as leading causes, especially in regions with high rates of malnutrition [23]. Drug-induced etiologies, such as chemotherapy or antiretroviral therapy, further expand the differential, particularly in patients undergoing treatment for chronic or infectious conditions [24]. VL was previously reported to be a cause of pancytopenia [25,26] and bicytopenia [27]. Our patient had low red blood cell and platelet count caused by *Leishmania* infection in the bone marrow, further highlighting the need of accurate diagnosis of bicytopenia.

The patient needed to go through repeated bone marrow aspirates to obtain a diagnosis. In the case of VL, initial bone marrow aspirates may fail to detect *Leishmania* parasites due to the low sensitivity of the procedure, which ranges between 60% and 85% [28]. Similarly, the Centers for Disease Control and Prevention (CDC) emphasizes the importance of repeating bone marrow aspirations or employing supplementary diagnostic methods, such as polymerase chain reaction, in cases where suspicion remains high despite negative results [29].

Our patient was diagnosed with VL after a kidney transplant due to an IgA nephropathy. The prevalence of VL in kidney transplant recipients varies but is primarily reported in endemic regions, including the Mediterranean basin, South America, and parts of Asia and Africa [30]. The onset of VL after kidney transplantation may vary significantly, often occurring months or years post-transplantation, either due to reactivation of latent infection or new acquisition through environmental exposure or, rarely, donor-derived transmission [30]. VL poses a significant risk to kidney transplant patients because it can lead to severe graft dysfunction due to parasitic infiltration, acute interstitial nephritis, and immune-mediated damage, with a mortality rate exceeding 20% if left untreated [31,32]. In our patient, it is unclear whether visceral leishmaniasis, in this case, was acquired before or after the transplant, but studies have shown that dormant *Leishmania* infections can reactivate following transplantation due to immunosuppression [33,34].

Similar to the signs and symptoms presented by our patient, clinical manifestations in kidney transplant recipients include fever, pancytopenia, hepatosplenomegaly, and graft dysfunction/loss. However, atypical presentations can be common due to the altered immune response [30,31]. Treatment with liposomal amphotericin B is preferred due to its efficacy and reduced nephrotoxicity compared to conventional therapies like pentavalent antimonials [31].

VL in Romania has been historically uncommon, with the first recorded case documented by Manicatide in 1912. A significant outbreak occurred in 1934, with 24 cases being reported in the Oltenia region [35]. Recently, a 28-year-old female from Sopot, Dolj County, contracted VL after traveling to Greece, underscoring the potential for imported cases in non-endemic regions [35]. Additionally, cutaneous leishmaniasis has been investigated in the region, revealing unique granulomatous responses, including a novel “messy granuloma” pattern characterized by disorganized histiocytes, described by Fernandez-Flores and Rodriguez-Peralto (2016) [36].

Our patient reported never leaving Cluj County and was the only organ recipient to present signs and symptoms due to leishmaniasis. A possible explanation is the presence of canine leishmaniasis in the region. Canine leishmaniasis, primarily caused by *Leishmania infantum*, poses a significant zoonotic threat due to dogs serving as the main reservoir for human visceral leishmaniasis [37]. Transmission to humans occurs through the bite of infected female phlebotomine sand flies, which acquire the parasite from infected dogs and subsequently transmit it to humans [38]. Mircean et al. reported an autochthonous clinical case in a dog from Valcea County (South-Central Romania) with severe symptoms and diagnosed with canine leishmaniasis [39]. In Ramnicu Valcea, the main city of Valcea County, a study conducted by Dumitrache et al. in 2016 detected *Leishmania infantum* in 7 (8.7%) of 80 dogs using conjunctival swab PCR, confirming an infection focus in the region [40]. Additionally, a study conducted in Arges County, a neighboring county of Valcea County, found that 30 (20.1%) of 149 asymptomatic kennel dogs tested positive for *Leishmania infantum* DNA, though all were seronegative for antibodies [41]. In 2018, Toma et al. reported a confirmed case of imported canine leishmaniasis in Cluj-Napoca, Romania. The affected dog, originally from Florence, Italy, was diagnosed through various tests, including complete blood cell count, serum biochemistry, cytology of lymph node aspirates and skin crusts, qualitative immunoassay, and real-time polymerase chain reaction. Due to the severity of the clinical signs and zoonotic risks, euthanasia was performed [42]. Studies have demonstrated that areas with a high prevalence of canine leishmaniasis often correspond with increased human leishmaniasis cases, highlighting the interconnectedness of canine and human disease dynamics [43]. Effective management of canine leishmaniasis, including monitoring and treating infected dogs, is crucial in mitigating the zoonotic transmission of leishmaniasis [44,45].

Climate also plays a critical role in the transmission of leishmaniasis, as environmental conditions influence the habitat and survival of sand fly vectors. Warmer temperatures, higher humidity, and changes in precipitation create optimal breeding conditions, enabling the spread of both vectors and reservoirs into new regions. These climate-driven shifts have been associated with the emergence of leishmaniasis in areas previously considered non-endemic, highlighting the need for ongoing environmental monitoring and vector control strategies [46].

Our patient received blood transfusions; in exceptional cases, leishmaniasis is transmitted through infected blood [12,47]. Currently, in Romania, no screening procedures for leishmaniasis are implemented in blood donors in Romania [48].

## 4. Conclusions

This report presents the first documented case of VL in a kidney transplant patient from Romania. Despite never leaving Cluj County and being the only organ recipient from the same donor to develop clinical signs and symptoms, the patient contracted the disease, suggesting a possible local source, which may be linked to canine leishmaniasis. This case also underscores the importance of considering leishmaniasis in immunosuppressed patients presenting with unexplained cytopenia, even in non-endemic areas. Moreover, this case highlighted the diagnostic challenges of relying on a single bone marrow aspirate, emphasizing the need for repeated sampling (aspirate and trephine biopsy) when the diagnosis may not be obvious. This case could serve as a starting point for future educational programs aimed at raising awareness among patients and healthcare professionals about the risk, diagnosis, and management of leishmaniasis in non-endemic regions.

## Figures and Tables

**Table 1:** Laboratory findings and progression in a 25-year-old renal transplant recipient with visceral leishmaniasis from Cluj County, Central Romania.

Parameter	Normal Values	Initial Presentation	Follow-Up Presentation
White Blood Count (WBC)	4–11 × 10^3^/µL	4.79 × 10^3^/µL	6.55 × 10^3^/µL
Neutrophils	40–60%	75.6%	69.9%
Lymphocytes	20–40%	17.5%	25.2%
Monocytes (MONO)	2–8%	6.7%	3.8%
Eosinophils	1–4%	0%	1.1%
Basophils (BASO)	0–1%	0.2%	0%
Red Blood Cell Count (RBC)	3.80–5.10 × 10^6^/µL	3.08 × 10^6^/µL	4.27 × 10^6^/µL
Hemoglobin (Hgl)	12.0–15.5 g/dL	9.1 g/dL	12.9 g/dL
Hematocrit (Hct)	34.9–44.5%	26.4%	35.8%
Mean Corpuscular Volume (MCV)	80–100 fL	85.7 fL	83.8 fL
Platelets (PLT)	150–450 × 10^3^/µL	18 × 10^3^/µL	105 × 10^3^/µL
Blood Glucose (BG)	70–110 mg/dL	123 mg/dL	97 mg/dL
Aspartate Transaminase (AST)	10–40 U/L	686 U/L	20 U/L
Alanine Transaminase (ALT)	7–56 U/L	829 U/L	31 U/L
Total Bilirubin	0.1–1.2 mg/dL	1.47 mg/dL	1.18 mg/dL
Direct Bilirubin	0–0.3 mg/dL	0.51 mg/dL	0.37 mg/dL
Lactate Dehydrogenase (LDH)	140–280 U/L	2084 U/L	453 U/L
Uric Acid	2.4–6.0 mg/dL	5.69 mg/dL	-
Alkaline Phosphatase (ALP)	30–120 U/L	340 U/L	-
Urea	10–50 mg/dL	42.6 mg/dL	-
Creatinine	0.5–1.0 mg/dL	1.04 mg/dL	-
Serum Calcium (Ca)	8.5–10.5 mg/dL	8.2 mg/dL	-
Serum Magnesium (Mg)	1.7–2.3 mg/dL	1.24 mg/dL	-
Procalcitonin (PCT)	<0.1 ng/mL	1.16 ng/mL	-
C-Reactive Protein (CRP)	<1 mg/dL	7.28 mg/dL	-
Ferritin	13–150 µg/L	934 µg/L	-

## Data Availability

The original contributions presented in this study are included in the article. Further inquiries can be directed to the corresponding authors.
